# Partisan differences in the effects of economic evidence and local data on legislator engagement with dissemination materials about behavioral health: a dissemination trial

**DOI:** 10.1186/s13012-022-01214-7

**Published:** 2022-06-22

**Authors:** Jonathan Purtle, Katherine L. Nelson, Luwam Gebrekristos, Félice Lê-Scherban, Sarah E. Gollust

**Affiliations:** 1 New York University School of Global Public Health, New York City, USA; 2 Drexel University Dornsife School of Public Health, Philadelphia, USA; 3 University of Minnesota School of Public Health, Minneapolis, USA

**Keywords:** Dissemination, Policymakers, Legislators, Adverse childhood experiences, Child maltreatment, Behavioral health, Politics, Partisanship, United States

## Abstract

**Background:**

State legislators make policy decisions that influence children’s exposure to adverse childhood experiences (ACEs), such as child maltreatment, and their effects on behavioral health. Effective dissemination of scientific research can increase the likelihood that legislators’ decisions are aligned with evidence to prevent ACEs and their consequences, and effective dissemination requires legislators to engage with dissemination materials. Informed by the elaboration likelihood model of persuasive communication and Brownson’s Model of Dissemination Research, we tested the hypothesis that inclusion of economic evidence and local data would increase legislator engagement with dissemination materials about evidence-supported policies related to ACEs and behavioral health.

**Methods:**

A three-arm randomized dissemination trial was conducted. A university researcher e-mailed dissemination materials which contained evidence about ACEs and behavioral health problems to state legislators (two e-mails sent 2 weeks apart, 12,662 e-mails delivered to 6509 legislators). The e-mail subject lines, text, and policy brief content were manipulated across the study arms. The intervention condition received state-tailored data about rates of ACEs and state-tailored economic evidence about the costs of ACEs for public systems, the enhanced control condition received state-tailored data and not economic evidence, and the control condition received national data and not economic evidence. Outcomes were rates of e-mail views, policy brief link clicks, requests for researcher consultation, and mentions of child maltreatment terms in legislators’ social media posts.

**Results:**

For the first e-mail, the e-mail view rate was 42.6% higher in the intervention than in the enhanced control condition (22.8% vs. 14.8%) and 20.8% higher than in the control condition (22.8% vs. 18.5%) (both *p* < .0001). Similar results were observed for the second e-mail. These differences remained significant after adjustment for demographic differences across study conditions in individual-level models, but not multilevel models. There was a significant interaction between the experimental condition and political party (*p* < .0001) in which the intervention increased e-mail view rates among Democrats but not Republicans. The intervention had no effect on policy brief link clicks or requests for consultation and a mixed effect on social media posts.

**Conclusions:**

Inclusion of state-tailored economic evidence in dissemination materials can increase engagement with research evidence among Democrat, but not Republican, legislators. Dissemination strategies tailored for legislators’ political party affiliation may be needed.

**Supplementary Information:**

The online version contains supplementary material available at 10.1186/s13012-022-01214-7.

Contributions to the literature

- Both dissemination research and policy-focused research are underdeveloped areas of implementation science, especially in the USA.
- Political identity has received little attention as a variable in implementation science.
- The current study illustrates how a cluster randomized design can be used to make causal inferences about the effects of including different types of evidence in dissemination materials for elected policymakers and the moderating effects of political identity.
- Inclusion of economic evidence in dissemination materials increases engagement with dissemination materials among Democrats, but not Republican, elected policymakers.
- Results support the inclusion of economic evidence in dissemination materials for elected policymakers, but also signal the potential value of tailoring dissemination materials for policymakers based on their political party affiliation.

## Background

Dissemination research—defined by the US National Institutes of Health as “the scientific study of targeted distribution of information and intervention materials to a specific public health or clinical practice audience” [1]—is underdeveloped in the field of implementation science [2, 3]. Policy-focused research is also an underdeveloped area of implementation science, particularly in the USA [4–6]. Thus, at the intersection of these two areas, it is not surprising that little empirical guidance exists to inform decisions about the dissemination of research evidence to policymakers in the USA. For example, a 2020 systematic review of strategies to disseminate research to US policymakers, published in *Implementation Science*, concluded that very little quantitative research has evaluated the effects of policymaker-targeted dissemination strategies [7]. As a consequence of this knowledge gap, dissemination decisions (e.g., decisions about which data to include in a policy brief or emphasize in e-mails to elected officials and their staff) are typically based on anecdote instead of evidence and have a suboptimal influence on policymaking [2, 8]. While capacity- and relationship-building strategies have demonstrated the ability to improve evidence-informed policymaking [9, 10], there is also a need to strengthen the empirical foundation for dissemination strategies that “push” evidence to policymakers because these strategies are far less resource intensive and are regularly used by researchers, advocates, and other intermediary organizations.

Building on descriptive research with state legislators about mental health and substance use (i.e., behavioral health) issues [11–18], the current study experimentally tests the effects of including state-tailored economic evidence and data in behavioral health dissemination materials on legislators’ engagement with dissemination materials. We also examine whether political party affiliation moderates the effects of dissemination materials and explore associations between political party affiliation and legislator engagement with dissemination materials. The study’s results provide concrete guidance to inform dissemination practice and the study’s design offers a model for future dissemination experiments with policymakers. The study also advances the field of implementation science by assessing the role of political identity in moderating dissemination outcomes, which has received little attention in prior work.

### Study context: evidence about adverse childhood experiences, behavioral health, and evidence-supported policy strategies

The current study focuses on disseminating evidence about adverse childhood experiences (ACEs) as risk factors for behavioral health problems and evidence-supported policy strategies to address ACEs. ACEs include incidents of child maltreatment—such as physical and sexual abuse and neglect—as well as other adverse experiences before age 18, such as witnessing domestic and community violence [19–22]. ACEs are well-established risk factors for behavioral health problems [22–24]. ACE exposure is common in the USA, with an estimated 16% of US adults having experienced ≥4 ACEs and 38% having experienced ≥2 ACEs [25].

State legislators (there are 7383 in the USA) are an important audience to target with the dissemination of evidence about ACEs because they make policy decisions that can reduce exposure to ACEs and mitigate their behavioral health consequences [26–28]. Reviews have identified state-level policies that have demonstrated effectiveness in achieving these outcomes [29–31]. These include policies that increase economic security and reduce caregiver stress [32–34] and policies that increase the reach of evidence-based public programs, such as nurse family partnerships [35, 36]. Organizations such as the American Academy of Pediatrics [37] and Harvard Center on the Developing Child [26] have emphasized the importance of disseminating evidence about ACEs to state legislators.

Despite the potential of state legislators to address ACEs, the legislative response to ACEs and legislators’ knowledge about ACEs are suboptimal. A recent review found that only 20.5% of bills introduced in state legislatures that mentioned ACEs also identified evidence-based or evidence-supported interventions related to ACEs [38]. This analysis also found that Democrats were significantly more likely than Republicans to introduce, co-sponsor, and vote in favor of legislation to address ACEs. A 2017 survey of 475 state legislators found that only one-third had heard of ACEs and that most were not knowledgeable about the extent to which ACEs were risk factors for behavioral health problems [14]. The survey also found that Republican legislators had significantly lower levels of knowledge about ACEs than Democrats. A nationally representative 2020 public opinion survey of US adults found similar results related to political party affiliation [39]. Public opinion surveys and experiments have also found, however, that evidence about the behavioral health consequences of ACEs and their economic impacts for public systems [40] could increase support for evidence-supported policies to address ACEs among both Republicans and Democrats [41, 42]. Taken together, this body of research underscores the importance of effectively disseminating evidence about ACEs to state legislators and provides some indication of messaging strategies that could be successfully used in practice.

### Prior dissemination research with state legislators and knowledge gap

Prior research conducted with state legislators provides additional guidance about how to develop dissemination materials about ACEs for state legislators. The aforementioned 2017 survey of legislators found that the local relevance of data and the inclusion of economic evidence were perceived as extremely important attributes of behavioral health dissemination materials, especially among Republicans [11, 13, 15]. The perceived importance of economic evidence and local data is consistent with a larger body of research about attributes of dissemination materials that are important to policymakers [7, 43–45]. Experiments with state and local legislators in the USA have also tested the effects of manipulating aspects of dissemination materials (e.g., framing of issues in e-mail subject lines [46–48], relational context [49, 50], inclusion of maps and narratives [51, 52]).

While prior work sheds light on how to format dissemination materials, it sheds little light on what *types of evidence to include*. This knowledge gap is important because only a finite amount of evidence can be included in concise dissemination materials, and research demonstrates that shorter presentations of evidence are preferred by legislators [2, 11]. Very little research has also explored the moderating effect of policymakers’ political party affiliation on the effects of dissemination materials [53]. This knowledge gap warrants attention given increasing political polarization among both state policymakers and the public in the USA [54, 55], the impact this polarization has on policymaking, and results from communication experiments with the general public which often find that political party identification moderates responses to different types of health messages [56, 57].

### Study aims and hypotheses

The primary aims of the study were to test the pre-registered hypotheses that:

- Inclusion of state-tailored economic evidence about ACEs *and* state-tailored data about rates of ACEs in dissemination materials (intervention) increases legislator engagement with dissemination materials compared with inclusion of national data about rates of ACEs and no economic evidence (control)
- Inclusion of state-tailored economic evidence about ACEs *and* state-tailored data about rates of ACEs in dissemination materials (intervention) increases engagement with dissemination materials compared with inclusion of state-tailored data about rates of ACEs and no economic evidence (enhanced control)
- Inclusion of state-tailored data about rates of ACEs and no economic evidence (enhanced control) increases engagement with dissemination materials compared with inclusion of national data about rates of ACEs and no economic evidence (control)

The secondary, exploratory aims were to determine whether the effects of dissemination materials were moderated by legislators’ political party affiliation and to assess the extent to which engagement with dissemination materials is independently associated with political party affiliation.

We focus on measures of engagement with dissemination materials as our outcome for three reasons. First, engagement is considered an important dissemination outcome [2, 58]. Measures of engagement with dissemination materials have been used in dissemination research conducted with policymakers [46–48] as well as clinicians [59] and are often treated as primary outcomes in experimental marketing research—a field that has similar goals as dissemination research [2, 60, 61]. Second, engagement can be assessed unobtrusively (i.e., without surveys or other research interaction) and this is important because it is increasingly challenging to obtain sufficiently high survey response rates from state legislators. Third, engagement with dissemination materials is a prerequisite for the materials to affect other dissemination outcomes such as knowledge, attitudes, and behavior change.

### Conceptual framework

The elaboration likelihood model (ELM) of persuasive communication provides the theoretical basis for our hypotheses [62–64]. ELM has been identified as a theory that has utility for dissemination research [2] and has been previously used in dissemination research with state legislators [48] and other practice audiences [65, 66]. ELM posits that the extent to which a message is perceived as *relevant* by audiences influences the extent to which audiences engage with and cognitively process the message. Based on prior research with state legislators [11, 13, 15], we believe that the inclusion of state-tailored economic evidence and data will increase the perceived relevance of dissemination materials and subsequent engagement with the materials. We further expand this model to consider the audience’s predisposing characteristics. Theories of motivated reasoning suggest that message acceptance and engagement is also dependent upon the extent to which the message resonates with an audience’s predisposing attitudes (i.e., attitudes about the relevance of or trust in scientific evidence) [67]. Given evidence of widening differences by partisanship in confidence in science, we examine legislator partisanship as a moderator [68].

The overarching structure of our study is also informed by Brownson and colleagues’ Model of Dissemination Research [3], which was used to guide the aforementioned systematic review of dissemination strategies targeting US policymakers [7]. To apply the Model to our study, the message *source* is a university researcher, the *message* is evidence about ACEs and behavioral health (which is experimentally manipulated), the *channel* used to deliver the message is e-mail, and the *audience* is US state legislators and their staff.

## Methods

### Study design, population, and randomization

We conducted a pre-registered, clustered randomized dissemination field experiment (Center for Open Science pre-registration: https://osf.io/cgh64; Supplemental file 1 CONSORT and TIDieR checklists). The study population was 6964 US state legislators (94% of those in the USA) who had an e-mail address in the KnowWho database as of January 25, 2021. KnowWho is a service that compiles and maintains up-to-date contact information and biographical data of government officials. We limit our sample and analyses to legislators for whom e-mail addresses were available because dissemination materials were delivered exclusively via e-mail. We refer to “legislators” throughout the article because they were our target audience, but it should be noted that we technically collected outcomes data from legislative offices because we do not know whether legislators or their staff engaged with dissemination materials.

Clustered, stratified randomization was used in which all fifty US states were randomized to one of the three conditions (detailed below). Randomization was at the state level to avoid contamination, wherein a legislator assigned to one condition would share dissemination materials with a legislator assigned to a different condition in the same state. Randomization was stratified to approximate balance in the number of legislators in each condition and the percentage of legislators in each condition that were Republican. We stratified on these two variables because the number of legislators in each state varies dramatically (i.e., range = 49 to 393) and prior research has shown that legislators’ opinions about ACEs vary significantly by political party [14].

To carry out stratified randomization, we classified each state according to whether the size of its legislature was less than the national median of 139 and whether the percentage of the legislature that was Republican was less than the national mean of 53.3%. This resulted in four strata of states defined by the cross-classification of legislature size and percentage of Republican legislators. We then randomized the states within each stratum for a balanced design, assigning all legislators within each state to the same study condition [69].

### Study conditions and dissemination materials

Legislators in the *intervention condition* were sent state-tailored data about rates of ACEs and state-tailored economic evidence about the costs of ACEs for public systems’ legislators in the *enhanced control condition* were sent state-tailored data about rates of ACEs and no economic evidence, and legislators in the control condition were sent national data about rates of ACEs and no economic evidence. Three elements of dissemination materials were manipulated in each condition: e-mail subject line, e-mail body test, and policy brief content.

The syntaxes of the e-mail subject lines were identical with the exception of elements that were modified to emphasize core aspects of each study condition. We used the language “Child Maltreatment,” as opposed to “Adverse Childhood Experiences,” in e-mail subject lines and language of “child maltreatment and adverse childhood experiences” in the e-mail body text because we assumed that more legislators would be familiar with the term “child maltreatment” than ACEs [14]. The subject lines for the three conditions are shown in Supplemental file 3.

The body text of the e-mails was modified similarly to summarize evidence contained in the policy brief (see Supplemental file 3 for e-mail text for all study conditions). Consistent with recommended practices to enhance e-mail engagement [61, 70], all e-mails were personalized to include the name and title of the legislator. Across all study conditions, the e-mails concluded with an invitation for consultation from the project principal investigator (PI) about evidence-supported policy approaches to addressing ACEs. This invitation was intended to provide relational context, which has been shown experimentally to increase policymaker engagement with research evidence [49, 50]. Furthermore, building relationships between researchers and policymakers is frequently identified as one of the most effective ways to facilitate more evidence use by policymakers [9, 44, 71, 72].

Policy briefs were accessible via a link in the e-mails that directed to a webpage where the policy brief was displayed in PDF format (see Supplemental file 4 for example policy briefs). Based on prior research demonstrating that state legislators have a strong preference for research evidence being concise [11], policy briefs were a single PDF page. Policy briefs across all three study conditions contained six identical elements:

- Descriptive information about types of experiences that are considered ACEs [19]
- Evidence about ACEs as risk factors for various behavioral health conditions in adulthood [23, 24, 73]
- A statement about the potential of positive childhood experience to prevent the consequences of ACEs [74]
- A statement about the COVID-19 pandemic having the potential to increase the incidence of ACEs [75–77]
- A list of evidence-supported state policies to address ACEs from the National Conference of State Legislators [29, 30], a professional association which is largely perceived as a credible source of research evidence among Democrat and Republican legislators [78]
- References for evidence cited

The policy briefs also included visual cues that corresponded with the study conditions [79]. The intervention condition policy brief included an image of a bank bag with a “$” to emphasize that economic evidence was included. The intervention and enhanced control condition policy briefs included an image of the legislator’s state to signal that state-tailored information was included. The control condition policy brief included an image of the USA.

The intervention and enhanced control policy briefs included state-tailored data about the prevalence of children in the state with ≥ 1 ACE and ≥ 2 ACEs, using data from the 2018 National Survey of Children’s Health, as well as data about the past-year incidence of reported cases of child maltreatment of any type, physical abuse, sexual abuse, and neglect—using data from the 2017 US National Child Abuse and Neglect Data System [80]. These types of child maltreatment are all considered ACEs [19–22]. In the control condition, the same data were presented at the US national level.

The intervention policy briefs included state-tailored economic evidence about the costs of some ACEs for state public systems. These estimates were generated by multiplying the number of reported incidents of non-fatal child maltreatment in each state in 2017 by an estimate of the lifetime cost of a single case of non-fatal child maltreatment—reported in a cost study by Peterson et al. [40] We calculated and included state-specific cost estimates from child welfare, special education, and criminal justice system perspectives (adjusted to be expressed in 2019 dollars) and presented this evidence in the policy briefs. We focused on costs from public system perspectives because prior research indicates that state legislators have a strong preference for economic evidence from this perspective [11].

### Dissemination procedure

Two e-mails were sent from the project PI’s university e-mail account using Qualtrics over the course of 3 weeks (first e-mail on March 22, 2021, second e-mail on April 5, 2021). Legislators for whom e-mails bounced back as undeliverable in the first e-mail (*n* = 492) were removed from the second e-mail.

### Outcomes and measures

The primary outcomes were legislator e-mail and policy briefs views. These were the primary outcomes because they were highly proximal indicators of engagement with dissemination materials. These outcomes were assessed using standard web-based marketing practices used in prior research [46–48, 59]. E-mail views were assessed by a 1-pixel translucent image that was embedded at the top of each e-mail. When an e-mail was viewed, the pixel was automatically downloaded, and the download was tracked in a de-identified database maintained by SSRS (a survey research firm). E-mail views were operationalized as a separate dichotomous outcome (yes/no) for each of the two e-mails. Policy brief views were assessed by monitoring link clicks to view the policy briefs. When a link was clicked, the legislator was automatically logged in to view the policy brief, and logins were tracked in the de-identified database maintained by SSRS. Policy brief views were also operationalized as a dichotomous outcome (yes/no) for each e-mail. Every legislator had a unique pixel ID and policy brief link for each e-mail, which allowed for these outcomes to be tracked at the individual level for the two separate e-mails.

Secondary outcomes were legislator requests for consultation about ACEs and mentions of topics related to ACEs in social media posts and newsletters to constituents. These were the secondary outcomes because they were more distal indicators of engagement with dissemination materials. E-mail replies and phone calls requesting consultation were tracked by the project PI and operationalized as a dichotomous outcome (yes/no) for each of the two e-mails. Mentions of topics related to ACEs in legislators’ Facebook and Twitter posts and newsletters to constituents were assessed using Quorum, a database that compiles this information in real time and has been previously used to characterize legislators’ public communication about health issues [81–83]. We searched Quorum for mentions of words from two sets of key terms in the 2-month period after when the first dissemination e-mail was sent (between March 22, 2021, and May 22, 2021). One set of terms was broadly related to the child maltreatment and the dissemination material content (i.e., “child abuse” and/or “physical abuse” and/or “child sexual abuse” and/or “child neglect” and/or “child maltreatment”). The other set of terms was explicitly related to ACEs (i.e., “adverse childhood experiences” and/or “ACEs”), the terms used in the aforementioned review of ACEs legislation [38].

### Covariates

Information about the demographic characteristics of legislators was obtained from the KnowWho database and served as covariates. Political party affiliation was operationalized as Republican or non-Republican (with non-Republicans hereafter referred to as Democrats because registered Democrats comprise over 98% of legislators in this category and consistent with how political party has been operationalized in prior work [15, 51]), gender was operationalized as male or female because this is a binary variable in the KnowWho database, and race/ethnicity was operationalized as non-Hispanic White (yes/no) to maximize statistical power. We also used KnowWho data to characterize each legislator according to whether they had a professional background in healthcare (yes/no).

### Analysis

Analysis was conducted at the individual legislator level because the e-mail dissemination intervention and outcome assessment were at this level. Legislators for whom e-mails were undeliverable (*n* = 492 in the first e-mail analysis, *n* = 356 in the second e-mail analysis) were excluded from the analysis. Data from the first and second dissemination e-mails were analyzed separately. Chi-square tests were used in randomization checks to assess differences in demographic characteristics of legislators across the three study conditions in each dissemination e-mail.

We use results from both unadjusted and adjusted analyses to determine the effects of dissemination materials because there is a lack of consensus about whether it is appropriate to adjust for demographic differences across conditions in randomized trials [84, 85]. We used chi-square tests to assess effects in unadjusted analyses and multivariable logistic regression models that adjusted for differences in demographic characteristics statistically significant at a threshold of *p* < .05. Legislators with missing data on covariates were excluded from regression analyses (*n* = 237 for the first e-mail, *n* = 213 for the second e-mail). We ran single as well as multilevel (legislator, state) random-intercept models to account for correlated outcomes between legislators in the same state (i.e., clustering) [86]. The intraclass correlation coefficient (ICC) ranged from 0.22 to 0.35 across the six main effect models (mean ICC across the models = 0.27), indicating that the data were suitable for multilevel analysis [87]. Our primary analytic approach was unadjusted because the sample size of our experiment was constrained by the number of state legislators in the USA and thus underpowered for multivariable models. We assessed whether the effects of dissemination materials were moderated by political party affiliation by stratifying results by political party within each study condition and by assessing the significance of a political party*study condition interaction term in regression models.

## Results

The first dissemination e-mail was successfully delivered to 6509 legislators and the second e-mail was delivered to 6153 legislators. CONSORT diagrams are included as Supplemental file 2. Table 1 shows the demographic characteristics of legislators to whom dissemination e-mails were successfully delivered, stratified across the three study conditions and two separate e-mails. There were small but statistically significant differences across the conditions in terms of political party affiliation, gender, and race/ethnicity.

**Table 1:** Demographics of US state legislators across the three study conditions, 2021

	Intervention		Enhanced control		Control		*P*
	*n*	%	*n*	%	*n*	%	
*First e-mail (n = 6509)*							
Political party							
Democrat	1167	48.6	1062	45.3	788	44.6	.02
Republican	1233	51.4	1281	54.7	978	55.4	
Gender							
Female	789	32.9	687	29.3	517	29.3	.01
Male	1610	67.1	1656	70.7	1248	70.7	
Health background							
Yes	93	4.2	100	4.7	86	5.0	.50
No	2098	95.8	2038	95.3	1622	95.0	
Race/ethnicity							
Non-Hispanic White	1967	85.2	1788	80.9	1360	77.5	<.0001
Not non-Hispanic White	341	14.8	422	19.1	394	22.5	
*Second e-mail (n = 6153)*							
Party							
Democrat	1164	48.7	954	47.6	782	44.5	.03
Republican	1227	51.3	1052	52.4	974	55.5	
Gender							
Female	785	32.9	597	29.8	514	29.3	.03
Male	1605	67.2	1409	70.2	1409	70.7	
Health background							
Yes	93	4.3	87	4.8	84	5.0	.57
No	2090	95.7	1744	95.2	1614	95.0	
Race/ethnicity							
Non-Hispanic White	1962	85.3	1499	79.1	1353	77.6	<.0001
Not non-Hispanic White	338	14.7	397	20.9	391	22.4	

### Effects of dissemination materials on e-mail views

E-mail views were registered for 1220 (18.7%) legislators in the first dissemination e-mail and 1089 (17.7%) in the second e-mail. In the first e-mail, the view rate was 42.6% higher in the intervention than enhanced control condition (22.8% vs. 14.9%) and 20.8% higher in the intervention than control condition (22.8% vs. 18.5%) (both *p* < .0001, Table 2). Similar effects were observed in the second dissemination e-mail, in which the view rate was 25.9% higher in the intervention than enhanced control condition (20.9% vs. 16.1%) and 31.6% higher in the intervention than control condition (20.9% vs. 15.2%) (both *p*<.0001).

**Table 2:** Unadjusted rates of engagement with dissemination materials among US state legislators, 2021

	E-mail view		Link click		Request for consultation	
	%	*p*	%	*p*	%	*p*
*First e-mail (n = 6509)*						
All	18.7	-	3.2	-	0.3	-
Condition						
Intervention	22.8	<.0001	3.3	.77	0.3	.98
Enhanced control	14.9		3.2		0.3	
Control	18.5		2.9		0.2	
Party						
Democrat	22.7	<.0001	4.5	<.0001	0.5	.001
Republican	15.3		2.1		0.1	
Gender						
Female	22.1	<.0001	5.7	<.0001	0.4	.25
Male	17.3		2.1		0.2	
Health background						
Yes	20.8	.42	4.7	.12	0.4	.65
No	18.8		3.0		0.2	
Race/ethnicity						
Non-Hispanic White	18.8	.99	3.2	.75	0.2	0.41
Not non-Hispanic White	18.8		3.0		0.4	
*Second e-mail (n = 6153)*						
All	17.7	-	2.3	-	0.4	-
Condition						
Intervention	20.9	<.0001	2.1	.21	0.3	0.93
Enhanced control	16.1		2.8		0.4	
Control	15.2		2.1		0.3	
Party						
Democrat	21.1	<.0001	3.1	<.0001	0.5	.05
Republican	14.7		1.6		0.2	
Gender						
Female	20.0	.002	3.7	<.0001	0.4	.57
Male	16.7		1.7		0.3	
Health background						
Yes	17.1	.69	2.3	.99	0.0	0.39
No	18.0		2.3		0.4	
Race/ethnicity						
Non-Hispanic White	17.7	.86	2.1	.37	0.3	0.09
Not non-Hispanic White	17.9		2.6		0.6	

The effect of the intervention treatment on e-mail views generally remained significant after adjustment for demographics in individual-level logistic regression models (Table 3). For example, compared to legislators in the control condition, the adjusted odds of a legislator in the intervention condition viewing the e-mail were 24% higher [adjusted odds ratio (AOR)=1.24; *p*= .008] in the first e-mail and 43% higher in the second e-mail (AOR= 1.43; *p* <.0001). The point estimates of the intervention effect were nearly identical in multilevel models that accounted for state clustering, but confidence intervals were wider and results were not statistically significant. The enhanced control treatment did not have a statistically significant effect on e-mail views compared to the control in unadjusted or adjusted analyses.

**Table 3:** Adjusted associations between study conditions, demographics, and engagement with dissemination materials among US state legislators, 2021

	Individual-level model						Multilevel model					
	E-mail view		Link click		Request for consultation		E-mail view		Link click		Request for consultation	
	AOR (95% CI)	*p*	AOR (95% CI)	*p*	AOR (95% CI)	*p*	AOR (95% CI)	*p*	AOR (95% CI)	*p*	AOR (95% CI)	*p*
*First e-mail (n = 6272)*												
Intervention vs. control (ref)	1.24 (1.06, 1.45)	.008	0.97 (0.67, 1.4)	.87	0.84 (0.22, 3.18)	.80	1.21 (0.55, 2.66)	.64	0.98 (0.51, 1.88)	.95	0.87 (0.19, 4.03)	.86
Intervention vs. enhanced control (ref)	1.64 (1.41, 1.91)	<.0001	0.92 (0.66, 1.28)	.61	0.74 (0.22, 2.43)	.62	1.47 (0.70, 3.13)	.31	0.94 (0.51, 1.74)	.85	0.67 (0.17, 2.68)	.57
Enhanced control vs. control	0.75 (0.64, 0.89)	.001	1.06 (0.74, 1.53)	.76	1.14 (0.32, 4.07)	.84	0.82 (0.37, 1.81)	.62	1.04 (0.54, 1.99)	.90	1.30 (0.30, 5.65)	.72
Democrat vs. Republican (ref)	1.71 (1.48, 1.98)	<.0001	2.09 (1.51, 2.90)	<.0001	8.37 (1.77, 39.65)	.007	1.49 (1.25, 1.76)	<.0001	2.13 (1.50, 3.00)	<.0001	8.59 (1.78, 41.48)	.007
Non-Hispanic White vs. not Non-Hispanic White (ref)	1.33 (1.11, 1.59)	.002	1.71 (1.16, 2.54)	.007	1.37 (0.42, 4.48)	.61	1.48 (1.19, 1.83)	.004	1.69 (1.12, 2.54)	.01	1.43 (0.43, 4.76)	.56
Female vs. male (ref)	1.16 (1.01, 1.34)	.03	2.48 (1.84, 3.35)	<.0001	0.97 (0.34, 2.77)	.95	1.18 (1.01, 1.38)	.04	2.50 (1.85, 3.39)	<.0001	0.98 (0.34, 2.81)	.96
*Second e-mail (n = 5940)*												
Intervention vs. control (ref)	1.43 (1.21, 1.69)	<.0001	0.91 (0.58, 1.42)	.67	1.02 (0.35, 2.98)	.97	1.47 (0.67, 3.2)	.34	0.91 (0.47, 1.75)	.77	1.02 (0.35, 2.98)	.97
Intervention vs. enhanced control (ref)	1.37 (1.17, 1.61)	<.0001	0.75 (0.50, 1.12)	0.16	0.97 (0.40, 2.70)	.95	1.18 (0.55, 2.53)	.68	0.82 (0.44, 1.55)	.55	0.97 (0.35, 2.70)	.95
Enhanced control vs. control (ref)	1.04 (0.87, 1.25)	.65	1.22 (0.78, 1.88)	.38	1.05 (0.35, 3.14)	.93	1.24 (0.56, 2.78)	.59	1.10 (0.57, 2.15)	.77	1.05 (0.35, 3.14)	.93
Democrat vs. Republican (ref)	1.65 (1.42, 1.92)	<.0001	2.07 (1.38, 3.1)	.0005	2.55 (0.88, 7.37)	.08	1.41 (1.17, 1.69)	.0002	1.94 (1.27, 2.95)	.002	2.55 (0.88, 7.37)	.08
Non-Hispanic White vs. not Non-Hispanic White (ref)	1.25 (1.04, 1.51)	.02	1.29 (0.83, 2.03)	.26	0.71 (0.26, 1.95)	.51	1.43 (1.14, 1.78)	.002	1.33 (0.83, 2.11)	.24	0.71 (0.26, 1.95)	.51
Female vs. male (ref)	1.09 (0.94, 1.27)	.24	1.88 (1.31, 2.7)	.0006	0.85 (0.34, 2.17)	.74	1.10 (0.93, 1.30)	.26	1.82 (1.26, 2.63)	.001	0.85 (0.34, 2.17)	.74

Multilevel models included a state-level random intercept. Models adjust for political party affiliation, race/ethnicity, and gender

*AOR* adjusted odds ratio

**Fig. 1:**
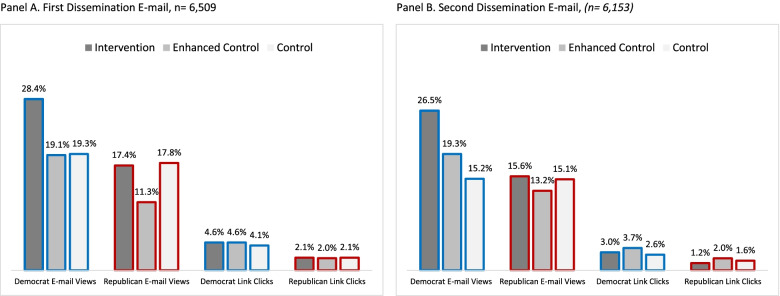
Unadjusted rates of engagement with dissemination materials among US state legislators stratified by political parity and study condition, 2021

The effects of the intervention treatment on e-mail views were moderated by political party affiliation. The political party*study condition interaction term was statistically significant in the individual-level model (intervention vs. control: *p*=. 002 and enhanced control vs. control: *p*= .0008). Among Democrats in the first e-mail, the e-mail view rate was 38.2% higher in the intervention than control condition (28.4% vs. 19.3%, *p* < .001), while the view rate was nearly identical among Republicans across these two conditions (17.4% vs. 17.8%) (Fig. 1). Similar results were observed in the second e-mail. Counter to our hypothesis, the e-mail view rate was significantly (*p* < .001) *lower* in the enhanced control than control group among Republicans in the first e-mail (11.3% vs. 17.8%) as well as the second e-mail (13.2% vs. 15.1%). This was not observed among Democrats.

### Intervention effects on policy brief link clicks

Policy brief link clicks were registered for 208 (3.2%) legislators in the first dissemination e-mail and 142 (2.3%) in the second e-mail. There were no significant differences in link click rates across the study conditions in unadjusted or adjusted analyses. There was no significant interaction between political party affiliation and study condition for link clicks.

### Intervention effects on requests for consultation

Following the first dissemination e-mail, 16 (0.3%) legislators requested consultation and 22 (0.4%) requested consultation following the second e-mail. There were no significant differences in the frequency of requests for consultation across the study conditions in unadjusted or adjusted analyses. There was also no significant interaction between political party affiliation and study condition for the consultation outcome.

### Intervention effects on mentions of terms related to ACEs in social media posts and newsletters to constituents

In the 2-month period following the date when the first dissemination e-mail was sent, 238 (3.9%) of legislators posted or sent ≥1 messages that mentioned any terms related to child maltreatment broadly or ACEs explicitly. The intervention condition did not impact the odds of a legislator mentioning the terms when mentions of the two terms were analyzed together (Table 4). Supplemental file 5 shows results from the separate analysis of mentions of terms broadly related to child maltreatment and terms explicitly related to ACEs. In these analyses, the intervention treatment did have the effect of significantly increasing the odds of a legislator mentioning a term broadly related to child maltreatment compared to the enhanced control group (AOR= 1.44, *p* = .04). However, the intervention treatment significantly reduced the odds of a legislator explicitly mentioning ACEs compared to the enhanced control (AOR= 0.10, *p* = .002) and control (AOR = 0.14, *p* = .0002) conditions.

**Table 4:** Unadjusted and adjusted associations between study conditions, demographics, and mentions of terms related to dissemination materials in US state legislators’ social media posts and newsletters to constituents, 2021

	Mention of any terms broadly related to child maltreatment or any explicit mention of ACEs					
	Unadjusted		Individual-level Model		Multilevel Model	
	OR (95% CI)	*p*	AOR (95% CI)	*p*	AOR (95% CI)	*p*
Intervention vs. control (ref)	0.99 (0.72, 1.34)	.93	0.98 (0.72, 1.35)	.91	0.90 (0.38, 2.12)	.80
Intervention vs. enhanced control (ref)	1.24 (0.90, 1.70)	.19	1.22 (0.88, 1.68)	.24	1.40 (0.59, 3.34)	.44
Enhanced control vs. control (ref)	0.80 (0.57, 1.12)	.19	0.81 (0.57, 1.14)	.23	0.64 (0.26, 1.58)	.33
Democrat vs. Republican (ref)	1.76 (1.35, 2.29)	<.0001	1.20 (0.88, 1.63)	.25	1.15 (0.83, 1.59)	.41
Non-Hispanic White vs. not non-Hispanic White (ref)	0.67 (0.50, 0.91)	.01	0.85 (0.60, 1.19)	.34	1.04 (0.73, 1.49)	.84
Female vs. male (ref)	2.88 (2.22, 3.74)	<.0001	2.62 (1.99, 3.46)	<.0001	2.75 (2.08, 3.66)	<.0001

Multilevel models included a state-level random intercept. Terms broadly related to child maltreatment = “child abuse” and/or “physical abuse” and/or “child sexual abuse” and/or “child neglect” and/or “child maltreatment”), explicit mentions of ACEs = “adverse childhood experiences” and/or “ACEs.” Models adjust for political party affiliation, race/ethnicity, and gender

*ACEs* adverse childhood experiences, *OR* odds ratio, unadjusted, *AOR* adjusted odds ratio

### Adjusted associations between political party affiliation and engagement with dissemination materials

After adjustment for study experimental condition, gender, and race/ethnicity, and state-level clustering, Democrats remained significantly more likely than Republicans to view the dissemination e-mails, view the policy briefs, and request consultation. For example, in the multilevel model for the first dissemination e-mail, Democrats had 49% higher odds of viewing the e-mail (AOR= 1.49 *p* < .0001), two times higher odds of clicking the policy brief link (AOR= 2.13, *p* < .0001), and over eight times higher odds of requesting consultation (AOR = 8.59, *p*= .007) than did Republicans. Similar results were observed in the second e-mail.

## Discussion

We experimentally tested the effects of including state-tailored economic evidence and state-tailored incidence/prevalence data on state legislator engagement with dissemination materials and explored associations between political party affiliation and engagement with dissemination materials. We found that the inclusion of economic evidence significantly increases engagement with the dissemination e-mails, but that this effect is entirely driven by Democrats. We did not find that the inclusion of economic evidence increased rates of clicking a link to view a policy brief or requesting expert consultation related to the dissemination materials. We also did not find that inclusion of state-tailored incidence/prevalence data increased engagement with dissemination materials compared to inclusion of national data. After adjustment for study condition and demographic covariates, we found that Democrats were significantly more likely to engage with dissemination materials than Republicans.

The significant effect of the intervention on e-mail view rates can be attributed to the mention of “economic impact” in the e-mail subject lines because viewing a subject line precedes viewing the body text of an e-mail. This finding is consistent with prior experiments which have found the e-mail subject lines influence e-mail view rates [46–48, 60, 61]. However, the magnitudes of the effects of subject lines on e-mail views in our experiment (e.g., 42.6% higher in the intervention than enhanced control group in the first e-mail) are larger than those observed in prior work. For example, a series of experiments with state legislators in 2020 which tested disparity vs. non-disparity frames in e-mail subject lines found effects on e-mail views that were no larger than 21% between study conditions [48]. A 2018 marketing experiment found that adding an e-mail recipient’s first name to the subject line increased the probability of viewing the e-mail by 20% [61]. According to the elaboration likelihood model of persuasive communication, the larger magnitudes of effects in our study may reflect economic evidence being perceived as highly relevant to state legislations, which is consistent with prior descriptive research with state legislators [13]. We did not find, however, that higher e-mail view rates in the intervention conditions translated into higher policy brief link click rates. These findings have at least two important implications for dissemination practice. First, results confirm that e-mail subject lines are an important element of dissemination materials and should be developed based on theory. Second, results highlight the importance of including the most important messages about evidence in the body text of dissemination e-mails because the majority of recipients who view an e-mail will not click a link to engage with additional evidence.

The finding that inclusion of state-tailored incidence/prevalence data did not increase engagement with dissemination materials compared to inclusion of national data is inconsistent with our hypothesis and some prior dissemination experiments with policymakers in the USA and the UK [88, 89]. Among Republicans, counter to our hypothesis, e-mail view rates were actually significantly *lower* among legislators who were sent state as opposed to national prevalence data. This finding warrants future investigation.

We found the inclusion of economic evidence had no effect on Republican legislators’ engagement with dissemination materials. This finding is inconsistent with our theoretically informed hypothesis and prior research which found that Republican legislators placed relatively more value on economic evidence related to behavioral health issues than Democrats [13, 15]. A recent survey-based experiment also observed results among Republican legislators that were counter to theory and prior research [53]. The experiment found that inclusion of narratives (i.e., fictional stories about people affected by the issue) decreased Republican legislators’ support for evidence-based childcare policies, while the narratives increased support among Democrats. In addition to the null effect of the intervention condition on Republican engagement with dissemination materials in our study, we found that Republican political party affiliation was significantly and inversely associated with all four of our engagement outcomes. Taken together, these findings signal a need for future research that tests the effects of tailoring messages in dissemination materials for Republican legislators and also potentially the sources from which they are delivered.

### Study key strengths and limitations

Key strengths of our study include the cluster randomized design (which enhances internal validity), assessment of outcomes via unobtrusive measures for the entire study population as opposed to surveys with a potentially non-representative sample of the population (which enhances external validity), assessment of multiple measures of engagement with dissemination materials ranging from the very proximal (i.e., e-mail views, policy brief link clicks) to more distal (i.e., requests for consultation, posts on social media), assessment outcomes at the individual level (e.g., tracking individual policy brief link clicks as opposed to aggregate policy brief website traffic), and the multilevel analysis approach.

A limitation of our study relates to the imprecise measurement of e-mail views and policy brief link clicks. Although these outcomes were assessed using marketing industry best practices that are standard in dissemination research, they are inherently imprecise because some e-mail servers and account settings block the automatic pixel downloads and link click logins used to register engagement outcomes. However, while this error may have affected study power, it should theoretically have been nondifferential with respect to study condition because of the study’s randomized design [90]. Our study was underpowered to detect significant effects in multilevel models. A post hoc power analysis revealed that, with 80% power for our primary outcomes, the minimally detectable odds would be 6.42 for the multilevel models. This cannot be addressed, however, because the number of state legislators and US states is fixed and nearly all (94%) were included in our study.

It should be emphasized that the e-mails were sent from a university researcher. While both Republican and Democrat legislators have identified university researchers as a credible source of research evidence in prior work [12], other studies have observed mistrust in researchers among legislators [78]. Our findings may have differed if dissemination materials were sent from another source. It should also be noted that there is no consensus, or even discussion, in the field in terms of what constitutes a “minimally important difference” in outcomes of engagement with dissemination materials. This is an important issue for building the field of dissemination research. Finally, our results are not necessarily generalizable beyond the US context. While some findings—such as the importance of e-mail subject lines—likely extend to different national contexts, the USA is unique in terms of its two-party political system and large number of states that have wide variation in staffing structure. E-mail is also a primary means of disseminating evidence to policymakers in the USA and this may not be the case in many low- and middle-income countries, limiting the generalizability of results to these contexts.

## Conclusion

Inclusion of state-tailored economic evidence in dissemination materials can increase engagement with research evidence among Democrat, but not Republican, legislators. Inclusion of state, as opposed to national, incidence/prevalence data does not have this effect, and national data might actually be *more* effective at prompting engagement with dissemination materials among Republican legislators. After adjustment for covariates, Republican legislators engaged with evidence disseminated from a university researcher at significantly lower rates than did Democrats. Taken together, the study findings support the inclusion of economic evidence in legislator-targeted dissemination materials but also highlight a need for future research that tests the effects of tailoring dissemination materials for legislators with different political party affiliations.

## Supplementary Information

**Additional file 1.** CONSORT and TIDieR checklists.

**Additional file 2.** CONSORT Diagram.

**Additional file 3.** E-mail text.

**Additional file 4.** Example Policy Briefs.

**Additional file 5.** Unadjusted and Adjusted Associations between Study Conditions, Demographics, and Mentions of Terms Related to Dissemination Materials in U.S. State Legislators’ Social Media Posts and Newsletters to Constituents, 2021.

## Data Availability

Data are however available from the authors upon reasonable request.
