# Supplementary material for: Partisan differences in the effects of economic evidence and local data on legislator engagement with dissemination materials about behavioral health: a dissemination trial
Source: Implement Sci. 2022 Jun 22;17:38. doi: 10.1186/s13012-022-01214-7 (PMC9213102; doi:10.1186/s13012-022-01214-7)

CONSORT

TRANSPARENT REPORTING of TRIALS

First E-Mail

Enrollment

Assessed for eligibility (N= 7,383)

Excluded (n= 419)
- No e-mail address in database (n= 3890)
- Vacant legislative seat (n= 29)

Randomized (n= 6,964)

Allocation

Allocated to intervention (n= 2,422)
- Received intervention (n= 2,400)
- Did not receive allocated intervention (e-mail bounce back to first policy brief) (n= 23)

Allocated to enhanced control (n= 2,358)
- Received intervention (n= 2,343)
- Did not receive allocated intervention (e-mail bounce back to first policy brief) (n= 14)

Allocated to control (n= 2,184)
- Received intervention (n= 1,766)
- Did not receive allocated intervention (e-mail bounce back to first policy brief) (n= 455)

Follow-Up

n/a

n/a

n/a

Analysis

Analysed (n= 2,400)
- Excluded from analysis (n= 0)

Analysed (n= 2,358)
- Excluded from analysis (n= 0)

Analysed (n= 1,756)
- Excluded from analysis (n= 0)

CONSORT
TRANSPARENT REPORTING of TRIALS

Second E-Mail

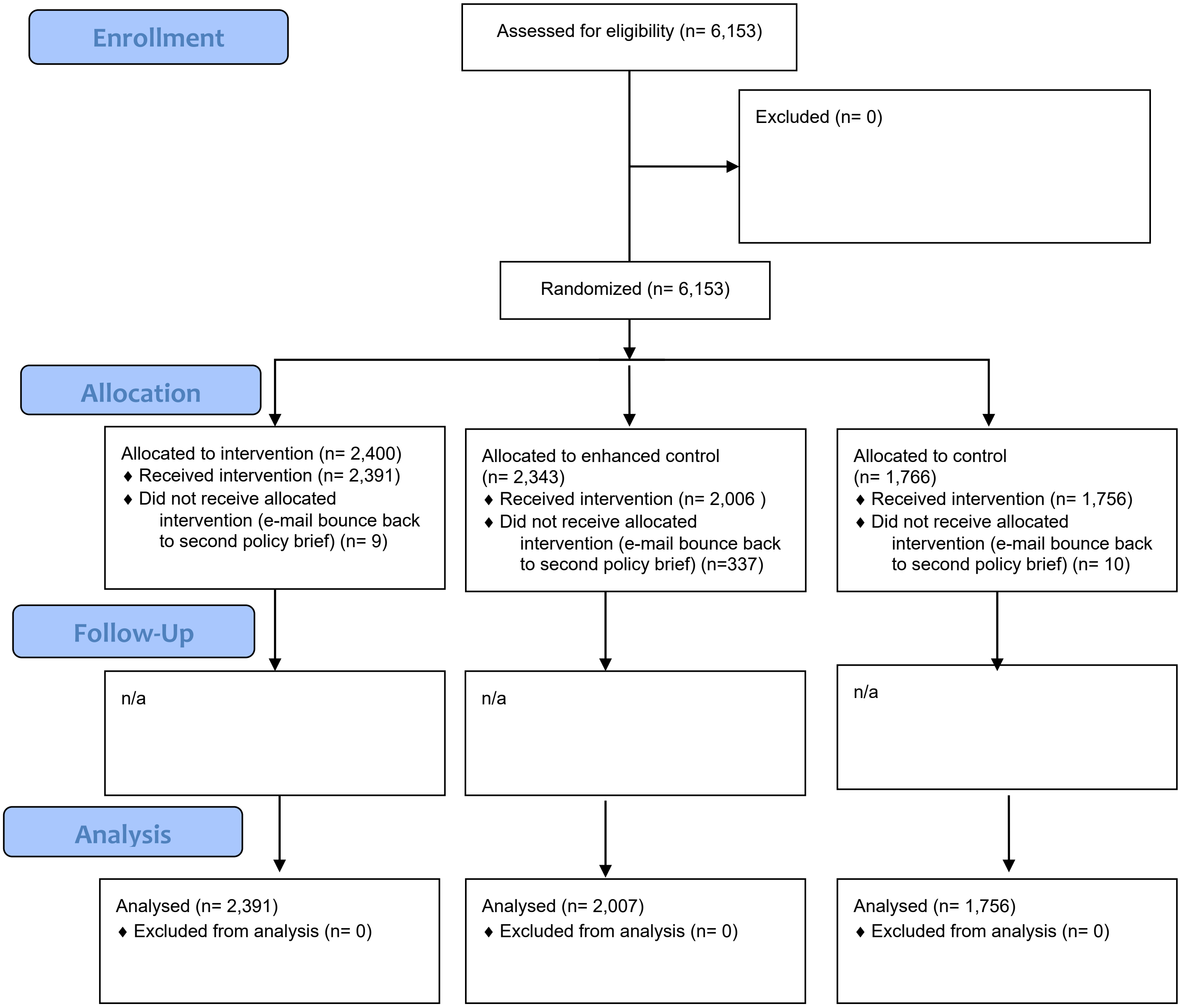

Supplement: Supplementary file 2 — Additional file 2. CONSORT Diagram. [file 13012_2022_1214_MOESM2_ESM.pdf]
