# Supplementary material for: Partisan differences in the effects of economic evidence and local data on legislator engagement with dissemination materials about behavioral health: a dissemination trial
Source: Implement Sci. 2022 Jun 22;17:38. doi: 10.1186/s13012-022-01214-7 (PMC9213102; doi:10.1186/s13012-022-01214-7)
Supplement: Supplementary file 3 — Additional file 3. E-mail text. [file 13012_2022_1214_MOESM3_ESM.pdf]

Intervention
Subject Child Maltreatment has Economic Impacts for ${e://Field/STATE}, Prevalent in ${e://Field/STATE}: Legislation Can Help

${e://Field/Unique_Pixal_Download_Link2}
Dear ${e://Field/PREFIX} ${e://Field/LASTNAME}:

I know that legislators receive countless e-mails and am thus re-sending the policy brief I sent two weeks ago. I'm a professor at the Drexel University School of Public Health and am leading a campaign to increase state legislator awareness about child maltreatment and strategies to prevent adult mental health and substance use problems. Data indicate that these issues are being exacerbated by the COVID-19 pandemic. I have developed a one-page policy brief about child maltreatment, adverse childhood experiences (ACEs), and their costs in ${e://Field/STATE}. The policy brief is available in PDF format here: ${e://Field/Policy_Brief_Link_2}. I can also send you the PDF directly if the link doesn't work.

The policy brief:
- Estimates of the economic impacts of child maltreatment and adverse childhood experiences for ${e://Field/STATE}'s public systems,
- Contains data about the annual number of cases of child maltreatment and adverse childhood experiences in ${e://Field/STATE},
- Summarizes evidence about the association between and adverse childhood experiences and adult mental health and substance use problems, and
- Highlights evidence-supported legislative approaches to addressing these issues.

As part of this awareness campaign, I'm also working with the survey research firm SSRS to field a survey that will collect information about how research findings can be more effectively communicated to state legislators and their staff. SSRS will be sending a link to this web-based survey next week.

I would be happy to discuss the policy brief with you or one of your staffers, or answer questions by e-mail. You can reach me by replying directly or by phone at the number below.

All the best,

Jonathan

Jonathan Purtle, DrPH, MSc
Associate Professor
Department of Health Management & Policy
Drexel University Dornsife School of Public Health
3215 Market St., Philadelphia, PA, 19104
Cell: 267-546-7541

${l://SurveyLink?d=}

Enhanced Control
Subject Child Maltreatment is Prevalent in ${e://Field/STATE}: Legislation Can Help
${e://Field/Unique_Pixal_Download_Link2}
Dear ${e://Field/PREFIX} ${e://Field/LASTNAME}:

I know that legislators receive countless e-mails and am thus re-sending the policy brief I sent two weeks ago. I'm a professor at the Drexel University School of Public Health and am leading a campaign to increase state legislator awareness about child maltreatment and strategies to prevent adult mental health and substance use problems. Data indicate that these issues are being exacerbated by the COVID-19 pandemic. I have developed a one-page policy brief about child maltreatment and adverse childhood experiences (ACEs) in ${e://Field/STATE}. The policy brief is available in PDF format here: ${e://Field/Policy_Brief_Link_2}. I can also send you the PDF directly if the link doesn't work.

The policy brief:

- Contains data about the annual number of cases of child maltreatment and adverse childhood experiences in ${e://Field/STATE},
- Summarizes evidence about the association between and adverse childhood experiences and adult mental health and substance use problems, and
- Highlights evidence-supported legislative approaches to addressing these issues.

As part of this awareness campaign, I'm also working with the survey research firm SSRS to field a survey that will collect information about how research findings can be more effectively communicated to state legislators and their staff. SSRS will be sending a link to this web-based survey next week.

I would be happy to discuss the policy brief with you or one of your staffers, or answer questions by e-mail. You can reach me by replying directly or by phone at the number below.

All the best,

Jonathan

Jonathan Purtle, DrPH, MSc
Associate Professor
Department of Health Management & Policy
Drexel University Dornsife School of Public Health
3215 Market St., Philadelphia, PA, 19104
Cell: 267-546-7541

${l://SurveyLink?d=}

Control
Subject Child Maltreatment is Prevalent in the United States: Legislation Can Help

${e://Field/Unique_Pixal_Download_Link2}
Dear ${e://Field/PREFIX} ${e://Field/LASTNAME}:

I know that legislators receive countless e-mails and am thus re-sending the policy brief I sent two weeks ago. I'm a professor at the Drexel University School of Public Health and am leading a campaign to increase state legislator awareness about child maltreatment and strategies to prevent adult mental health and substance use problems. Data Indicate that these issues are being exacerbated by the COVID-19 pandemic. I have developed a one-page policy brief about child maltreatment and adverse childhood experiences (ACEs) in the United States. The policy brief is available in PDF format here: ${e://Field/Policy_Brief_Link_2}. I can also send you the PDF directly if the link doesn't work.

The policy brief:

- Contains data about the annual number of cases of child maltreatment and adverse childhood experiences in the United States,
- Summarizes evidence about the association between and adverse childhood experiences and adult mental health and substance use problems, and
- Highlights evidence-supported legislative approaches to addressing these issues.

As part of this awareness campaign, I'm also working with the survey research firm SSRS to field a survey that will collect information about how research findings can be more effectively communicated to state legislators and their staff. SSRS will be sending a link to this web-based survey next week.

I would be happy to discuss the policy brief with you or one of your staffers, or answer questions by e-mail. You can reach me by replying directly or by phone at the number below.

All the best,

Jonathan

Jonathan Purtle, DrPH, MSc
Associate Professor
Department of Health Management & Policy
Drexel University Dornsife School of Public Health
3215 Market St., Philadelphia, PA, 19104
Cell: 267-546-7541

${l://SurveyLink?d=}
