# Supplementary material for: Partisan differences in the effects of economic evidence and local data on legislator engagement with dissemination materials about behavioral health: a dissemination trial
Source: Implement Sci. 2022 Jun 22;17:38. doi: 10.1186/s13012-022-01214-7 (PMC9213102; doi:10.1186/s13012-022-01214-7)
Supplement: Supplementary file 4 — Additional file 4. Example Policy Briefs. [file 13012_2022_1214_MOESM4_ESM.pdf]

Adverse Childhood Experiences in Arkansas:
Costs and Mental Health/Substance Use Impacts

What are Adverse Childhood Experiences (ACEs)?

Adverse childhood experiences (ACEs) include things like abuse and neglect, having a parent with a serious substance use disorder or mental illness, and witnessing domestic or community violence. Studies have repeatedly shown that the risk of having mental health or substance use problems as an adult increases with the number of ACEs that a person has had. Positive childhood experiences can also prevent the consequences of ACEs (1). Research suggests that the social and economic consequences of the COVID-19 pandemic could result in more ACEs.

ACEs Increase Risk for Adult Mental Health and Substance Use Problems (2)

Compared to adults with 0 ACEs, adults with 4 or more ACEs have:

- 30.1 times higher odds of attempting suicide
- 10.2 times higher odds of problematic drug use
- 5.8 times higher odds of problematic alcohol use
- 4.4 times higher odds of depression

Percentage of Mental Health and Substance Use Problems in the U.S. Attributable to ACEs (3)

Depression 44%

Heavy Drinking 24%

ACEs are Common and Costly in Arkansas (4)

In Arkansas in 2017 there were:

- 9,334 cases of child maltreatment reported
  - 2,051 cases of child physical abuse
  - 1,783 cases of child sexual abuse
  - 5,043 cases of child neglect

Lifetime Public Systems Costs Caused by Cases of Child Maltreatment in Arkansas, 2017 (4,5)

Child Welfare: $78,396,266
Special Education: $81,140,462
Criminal Justice: $68,446,222

State Laws Can Prevent ACEs and their Consequences (7)

The National Conference of State Legislators summarized evidence about state laws that work. These include:

- Increasing access to early childhood education
- Expanding school-based mental health services
- Raising the minimum wage, extending earned income tax credits
- Funding nurse-family partnerships
- Increasing access to treatments than are proven to help people recover from mental health and substance use conditions

Percentage of Children in Arkansas with ACEs, Parent Reported, 2018 (6)

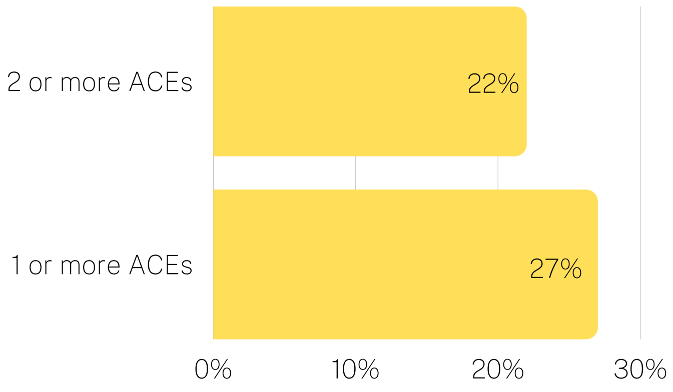

More Information and References:

1. Bethell et al. Positive childhood experiences and adult mental and relational health in a statewide sample. *JAMA Pediatrics*. 2019.
2. Hughes et al. The effect of multiple adverse childhood experiences on health: *The Lancet Public Health*. 2017.
3. Merrick et al. Estimated Proportion of Adult Health Problems Attributable to Adverse Childhood Experiences. *MMWR*. 2019.
4. US Department of Health and Human Services. *Child Maltreatment*. 2017. https://bit.ly/2Z7IgGZ
5. Peterson et al. The economic burden of child maltreatment in the United States, 2015. *Child Abuse & Neglect*. 2018
6. National Survey of Children's Health. 2018 https://www.childhealthdata.org/browse/survey/allstates?q=7442
7. NCSL. Preventing and Mitigating the Effects of Adverse Childhood Experiences. 2018. https://bit.ly/38U6JEr

Questions?

Evidence brief by:
Jonathan Purtle, DrPH
Associate Professor
Drexel University
School of Public Health
E-mail: JPP46@Drexel.edu
Phone: 267-546-7541

Adverse Childhood Experiences in Alabama:
Mental Health/Substance Use Impacts

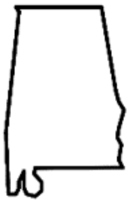

What are Adverse Childhood Experiences (ACEs)?

Adverse childhood experiences (ACEs) include things like abuse and neglect, having a parent with a serious substance use disorder or mental illness, and witnessing domestic or community violence. Studies have repeatedly shown that the risk of having mental health or substance use problems as an adult increases with the number of ACEs that a person has had. Positive childhood experiences can also prevent the consequences of ACEs (1). Research suggests that the social and economic consequences of the COVID-19 pandemic could result in more ACEs.

ACEs Increase Risk for Adult Mental Health and Substance Use Problems (2)

Compared to adults with 0 ACEs, adults with 4 or more ACEs have:

- 30.1 times higher odds of attempting suicide
- 10.2 times higher odds of problematic drug use
- 5.8 times higher odds of problematic alcohol use
- 4.4 times higher odds of depression

ACEs are Common and Costly in Alabama (4)

In Alabama in 2017 there were:

- 10,847 cases of child maltreatment reported
  - 5,720 cases of child physical abuse
  - 1,590 cases of child sexual abuse
  - 4,669 cases of child neglect

State Laws Can Prevent ACEs and their Consequences (6)

The National Conference of State Legislators summarized evidence about state laws that work. These include:

- Increasing access to early childhood education
- Expanding school-based mental health services
- Raising the minimum wage, extending earned income tax credits
- Funding nurse-family partnerships
- Increasing access to treatments than are proven to help people recover from mental health and substance use conditions

Percentage of Mental Health and Substance Use Problems in the U.S. Attributable to ACEs (3)

Depression 44%

Heavy Drinking 24%

Percentage of Children in Alabama with ACEs, Parent Reported, 2018 (5)

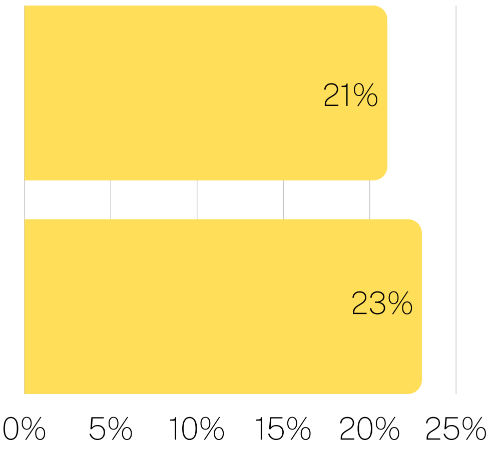

More Information and References:

1. Bethell et al. Positive childhood experiences and adult mental and relational health in a statewide sample. *JAMA Pediatrics.* 2019.
2. Hughes et al. The effect of multiple adverse childhood experiences on health: *The Lancet Public Health.* 2017.
3. Merrick et al. Estimated Proportion of Adult Health Problems Attributable to Adverse Childhood Experiences. *MMWR.* 2019.
4. US Department of Health and Human Services. *Child Maltreatment.* 2017. https://bit.ly/2Z7IgGZ
5. National Survey of Children's Health. 2018 https://www.childhealthdata.org/browse/survey/allstates?q=7442
6. NCSL. Preventing and Mitigating the Effects of Adverse Childhood Experiences. 2018. https://bit.ly/38U6JEr

Questions?
Evidence brief by:
Jonathan Purtle, DrPH
Associate Professor
Drexel University
School of Public Health
E-mail: JPP46@Drexel.edu
Phone: 267-546-7541

Adverse Childhood Experiences in the US:

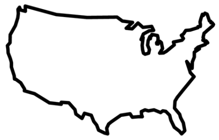

Mental Health/Substance Use Impacts

What are Adverse Childhood Experiences (ACEs)?

Adverse childhood experiences (ACEs) include things like abuse and neglect, having a parent with a serious substance use disorder or mental illness, and witnessing domestic or community violence. Studies have repeatedly shown that the risk of having mental health or substance use problems as an adult increases with the number of ACEs that a person has had. Positive childhood experiences can also prevent the consequences of ACEs (1). Research suggests that the social and economic consequences of the COVID-19 pandemic could result in more ACEs.

ACEs Increase Risk for Adult Mental Health and Substance Use Problems (2)

Compared to adults with 0 ACEs, adults with 4 or more ACEs have:

- 30.1 times higher odds of attempting suicide
- 10.2 times higher odds of problematic drug use
- 5.8 times higher odds of problematic alcohol use
- 4.4 times higher odds of depression

ACEs are Common and Costly in the US (4)

In the USA in 2017 there were:

- 673,830 cases of child maltreatment reported
  - 123,065 cases of child physical abuse
  - 58,114 cases of child sexual abuse
  - 504,545 cases of child neglect

State Laws Can Prevent ACEs and their Consequences (6)

The National Conference of State Legislators summarized evidence about state laws that work. These include:

- Increasing access to early childhood education
- Expanding school-based mental health services
- Raising the minimum wage, extending earned income tax credits
- Funding nurse-family partnerships
- Increasing access to treatments than are proven to help people recover from mental health and substance use conditions

Percentage of Mental Health and Substance Use Problems in the U.S. Attributable to ACEs (3)

Depression 44%

Heavy Drinking 24%

Percentage of Children in the US with ACEs, Parent Reported, 2018 (5)

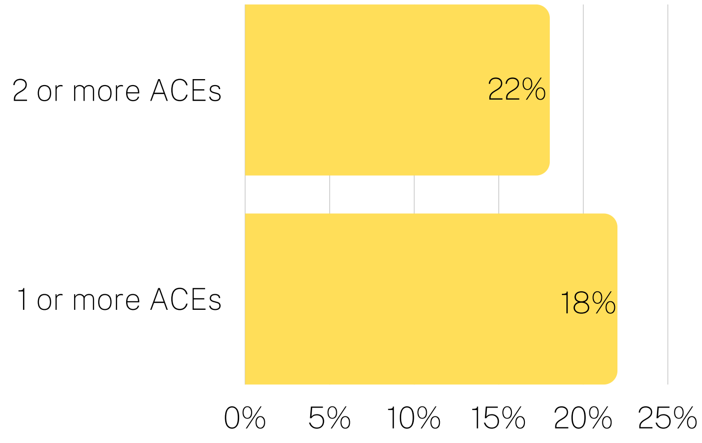

More Information and References:

1. Bethell. et al. Positive childhood experiences and adult mental and relational health in a statewide sample. *JAMA Pediatrics*. 2019.
2. Hughes et al. The effect of multiple adverse childhood experiences on health: *The Lancet Public Health*. 2017.
3. Merrick et al. Estimated Proportion of Adult Health Problems Attributable to Adverse Childhood Experiences. *MMWR*. 2019.
4. US Department of Health and Human Services. *Child Maltreatment*. 2017. https://bit.ly/2Z7lgGZ
5. National Survey of Children's Health. 2018. https://www.childhealthdata.org/browse/survey/allstates?q=7442
6. NCSL. Preventing and Mitigating the Effects of Adverse Childhood Experiences. 2018. https://bit.ly/38U6JEr

Questions?

Evidence brief by:
Jonathan Purtle, DrPH
Associate Professor
Drexel University
School of Public Health
E-mail: JPP46@Drexel.edu
Phone: 267-546-7541
