# Supplementary material for: Partisan differences in the effects of economic evidence and local data on legislator engagement with dissemination materials about behavioral health: a dissemination trial
Source: Implement Sci. 2022 Jun 22;17:38. doi: 10.1186/s13012-022-01214-7 (PMC9213102; doi:10.1186/s13012-022-01214-7)
Supplement: Supplementary file 5 — Additional file 5. Unadjusted and Adjusted Associations between Study Conditions, Demographics, and Mentions of Terms Related to Dissemination Materials in U.S. State Legislators’ Social Media Posts and Newsletters to Constituents, 2021. [file 13012_2022_1214_MOESM5_ESM.docx]

**Supplemental File 5: Unadjusted and Adjusted Associations between Study Conditions, Demographics, and Mentions of Terms Related to Dissemination Materials in U.S. State Legislators’ Social Media Posts and Newsletters to Constituents, 2021**

|  | Unadjusted | | | | Individual-level Model | | | | Multilevel Model | | | |
| --- | --- | --- | --- | --- | --- | --- | --- | --- | --- | --- | --- | --- |
|  | Mention of Terms Related to Child Maltreatment | | Explicit Mention of Adverse Childhood Experiences | | Mention of Terms Related to Child Maltreatment | | Explicit Mention of Adverse Childhood Experiences | | Mention of Terms Related to Child Maltreatment | | Explicit Mention of Adverse Childhood Experiences | |
|  | OR | *p* | OR | *p* | AOR | *p* | AOR | *p* | AOR | *p* | AOR | *p* |
| Intervention vs. Control (ref) | 1.13 (0.82,1.57) | .46 | 0.21 (0.07,0.64) | .006 | 1.15 (0.82,1.60) | .42 | 0.14 (0.04,0.48) | .002 | 1.08 (0.44,2.64) | .86 | 0.17 (0.03,0.92) | .04 |
| Intervention vs.  Enhanced Control (ref) | 1.44 (1.03,2.02) | .03 | 0.14 (0.05,0.42) | .0004 | 1.44 (1.02,2.04) | 0.04 | 0.10 (0.03,0.33) | .0002 | 1.51 (0.62,3.68) | .37 | 0.25  (0.04, 1.43) | .12 |
| Enhanced Control vs. Control (ref) | 0.79 (0.54,1.13) | .20 | 1.44 (0.74,2.81) | .28 | 0.79 (0.55,1.16) | .22 | 1.41 (0.72,2.77) | .31 | 0.72 (2.28,1.84) | .49 | 0.67 (0.16,2.79) | .58 |
| Democrat vs.  Republican (ref) | 1.56 (1.18,2.05) | .002 | 5.50 (2.44,12.43) | <.0001 | 1.04 (0.75,1.44) | .80 | 4.76 (1.98,11.42) | .0005 | 1.03 (0.73,1.45) | .89 | 3.63 (1.48,8.91) | .005 |
| Non-Hispanic White vs. Not Non-Hispanic White (ref) | 0.73 (0.53,1.01) | .06 | 0.61 (0.31,1.23) | .17 | 0.85 (0.59,1.22) | .38 | 1.45 (0.70,3.10) | .32 | 1.02  (0.70, 1.50) | .90 | 1.86 (0.87,3.97) | .11 |
| Female vs.  Male (ref) | 2.91 (2.21,3.83) | <.0001 | 3.54 (1.89,6.65) | <.0001 | 2.76 (2.06,3.69) | <.0001 | 2.50 (1.30,4.83) | .006 | 2.87 (2.13,3.87) | <.0001 | 2.93 (1.49,5.76) | .002 |

OR= odds ratio, unadjusted; AOR= adjusted odds ratio. Multilevel models included a state-level random intercept. Models adjust for political party affiliation, race/ethnicity, and gender.
